# The rice *GERMINATION DEFECTIVE 1*, encoding a B3 domain transcriptional repressor, regulates seed germination and seedling development by integrating GA and carbohydrate metabolism

**DOI:** 10.1111/tpj.12209

**Published:** 2013-05-13

**Authors:** Xiaoli Guo, Xiaomei Hou, Jun Fang, Piwei Wei, Bo Xu, Mingluan Chen, Yuqi Feng, Chengcai Chu

**Affiliations:** 1 State Key Laboratory of Plant Genomics and National Center for Plant Gene Research (Beijing), Institute of Genetics and Developmental Biology, Chinese Academy of SciencesBeijing, 100101, China; 2 Graduate University of the Chinese Academy of SciencesBeijing, 100049, China; 3 Key Laboratory of Analytical Chemistry for Biology and Medicine (Ministry of Education) Department of Chemistry, Wuhan UniversityWuhan, 430072, China

**Keywords:** B3 domain transcription factor, EAR motif, *germination-defective1*, gibberellin, rice, seed germination

## Abstract

It has been shown that seed development is regulated by a network of transcription factors in Arabidopsis including LEC1 (LEAFY COTYLEDON1), L1L (LEC1-like) and the B3 domain factors LEC2, FUS3 (FUSCA3) and ABI3 (ABA-INSENSITIVE3); however, molecular and genetic regulation of seed development in cereals is poorly understood. To understand seed development and seed germination in cereals, a large-scale screen was performed using our T–DNA mutant population, and a mutant *germination-defective1* (*gd1*) was identified. In addition to the severe germination defect, the *gd1* mutant also shows a dwarf phenotype and abnormal flower development. Molecular and biochemical analyses revealed that *GD1* encodes a B3 domain-containing transcription factor with repression activity. Consistent with the dwarf phenotype of *gd1*, expression of the gibberelic acid (GA) inactivation gene *OsGA2ox3* is increased dramatically, accompanied by reduced expression of GA biosynthetic genes including *OsGA20ox1*, *OsGA20ox2* and *OsGA3ox2* in *gd1*, resulting in a decreased endogenous GA_4_ level. Exogenous application of GA not only induced *GD1* expression, but also partially rescued the dwarf phenotype of *gd1*. Furthermore, GD1 binds to the promoter of *OsLFL1*, a *LEC2/FUS3*-like gene of rice, via an RY element, leading to significant up-regulation of *OsLFL1* and a large subset of seed maturation genes in the *gd1* mutant. Plants over-expressing *OsLFL1* partly mimic the *gd1* mutant. In addition, expression of *GD1* was induced under sugar treatment, and the contents of starch and soluble sugar are altered in the *gd1* mutant. These data indicate that GD1 participates directly or indirectly in regulating GA and carbohydrate homeostasis, and further regulates rice seed germination and seedling development.

## Introduction

Seed development is an important phase in the life cycle of land plants, offering plants an indispensable opportunity to maintain the species and to survive in a hostile environment. In flowering plants, seed development may be divided into two major stages: embryo/endosperm morphogenesis and seed maturation. A large body of evidence obtained over recent years has shown that many genetic loci control the developmental regulation from embryogenesis to germination (McCarty, 1995; Holdsworth *et al*., 1999). It has been extensively demonstrated that LEAFY COTYLEDON 1 (LEC1), LEC2, FUSCA3 (FUS3) and ABSCISIC ACID INSENSITIVE 3 (ABI3) play a central role in this process. *LEC1* encodes a conserved eukaryotic CCAAT-box binding HAP3 subunit, while *LEC2*, *FUS3* and *ABI3* all encode proteins with a plant-specific B3 DNA-binding domain (Giraudat *et al*., 1992; Lotan *et al*., 1998; Luerssen *et al*., 1998; Stone *et al*., 2001). The four mutants *lec1*, *lec2*, *fus3* and *abi3* severely block the seed development and share some common phenotypes, such as reduced expression of seed storage proteins (Kroj *et al*., 2003). By contrast, enforced expression of *LEC1* or *LEC2* is sufficient to induce embryo development in vegetative cells (Lotan *et al*., 1998; Stone *et al*., 2001). LEC2 represses expression of *GA3ox2* and may regulate embryogenesis partly by stimulating expression of auxin biosynthesis genes (Stone *et al*., 2001, 2008; Curaba *et al*., 2004; Braybrook *et al*., 2006). Restriction of expression of *FUS3* to the epidermis produces cotyledon-like leaves, and *FUS3* negatively modulates GA biosynthesis and positively regulates abscisic acid (ABA) biosynthesis (Gazzarrini *et al*., 2004). *GA3ox2* is also a direct downstream target of *FUS3* (Curaba *et al*., 2004). These loci may act synergistically to adjust the optimal hormone balance to maintain seed development (To *et al*., 2006).

Recently, a striking functional symmetry within the B3 domain-containing transcription factors was revealed by identification of a novel B3 domain transcriptional repressor named VP1/ABI3-LIKE (VAL; Suzuki and McCarty, 2008). In Arabidopsis, three genes (*VAL1*, *VAL2* and *VAL3*) are categorized within the VAL sub-group. In addition to a B3 domain, the VAL proteins contain three other domains that may be involved in chromatin remodeling: a PHD (plant homeodomain)-like domain, a CW-type zinc finger, and an EAR (ethylene response factor-associated amphiphilic repression) motif. The *val1* mutant was first identified by screening for mutants with altered expression of sugar response genes (Tsukagoshi *et al*., 2005). The *val1*, *val2* and *val3* single mutants have no obvious phenotype compared to the wild-type under normal growth conditions, while the *val1* mutant exhibits strong embryonic traits when germinated on medium containing paclobutrazol, an inhibitor of GA biosynthesis (Suzuki *et al*., 2007). However, the *val1 val2* double mutant and *val1 val2 val3* triple mutant seedlings show growth arrest and develop a variety of embryonic phenotypes during their growth and development (Suzuki *et al*., 2007). Expression of *LEC1*, *LEC2*, *FUS3*, *ABI3* and other seed maturation genes was up-regulated in the *val1 val2* double mutant, suggesting that *VAL* genes play a role in repressing the embryonic pathway and are essential for the transition from embryo development to seed germination and vegetative development (Suzuki *et al*., 2007). These findings in Arabidopsis reveal that two sub-families of B3 domain transcription factors serve as activators and repressors of seed development, respectively, but the mechanism of how those regulators are involved in the seed development is still unclear. In contrast to ABI3, FUS3 and LEC2, which have well-defined *cis*-elements in their downstream target genes, *cis*-elements specific to VAL transcription factors remain to be elucidated (Suzuki *et al*., 1997; Ezcurra *et al*., 2000; Reidt *et al*., 2000; Monke *et al*., 2004; Braybrook *et al*., 2006). VAL transcription factors may target RY elements (typical sequence CATGCATG) for repression through recruitment of chromatin modification and remodeling factors (Suzuki *et al*., 2007; Guerriero *et al*., 2009; Kagale and Rozwadowski, 2011).

In rice (*Oryza sativa*), it was found that 91 B3 domain-containing genes exist (Swaminathan *et al*., 2008), but *OsLFL1* (*LEC2*/*FUS3-LIKE*), which is most close to *FUS3* in Arabidopsis, is the only rice B3 gene with a phenotype reported to date (Swaminathan *et al*., 2008). It has been shown that OsLFL1 binds directly to the RY motif in the *Early heading date 1* (*Ehd1*) promoter, and over-expression of *OsLFL1* delays flowering by repressing *Ehd1* expression (Peng *et al*., 2007, 2008). In addition to late-flowering, additional phenotypes such as dwarf stature, small leaves and leaf-like petals were also observed on over-expression of *OsLFL1* in rice and Arabidopsis, suggesting that *OsLFL1* may have a similar function to *LEC2* and *FUS3* of Arabidopsis (Peng *et al*., 2007, 2008). In cereals, the correct transition from embryo development to germination is an important agronomic trait, but little is known about the molecular and genetic regulation of this process (Holdsworth *et al*., 1999). Here, we report the identification and characterization of a rice mutant *germination-defective1* (*gd1*), which is defective in seed germination and seedling development. When imbibed in water, some of the mutants arrested during germination period. Once germinated, the growth rate of *gd1* is also much slower than that of wild-type. Sequence alignment showed that GD1 has high similarity to VALs in Arabidopsis. Further molecular and biochemical analyses revealed that GD1 has transcriptional repressor activity and ability to bind the RY element. In addition, plants over-expressing *OsLFL1* partly mimic the *gd1* mutant. The data clearly indicate that GD1 participates directly or indirectly in GA homeostasis by suppressing *OsLFL1* expression. These results extend our understanding on the role of B3 genes in regulating rice seed germination and seedling development.

## Results

### Phenotype of *germination-defective1*

**Figure 1:**
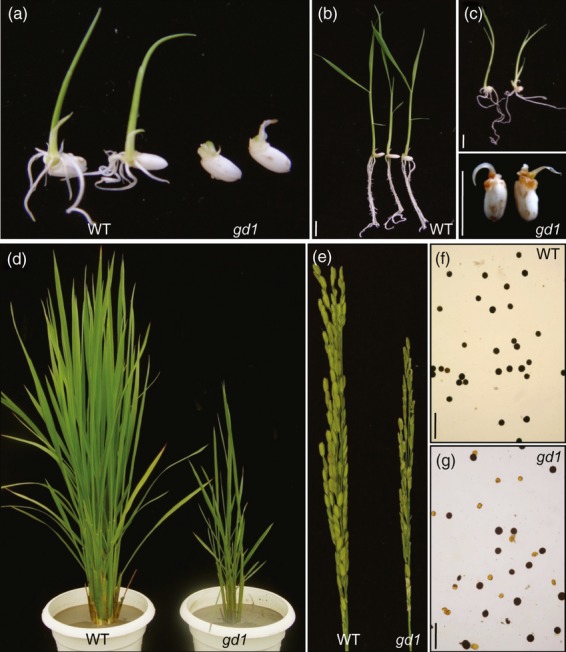
Phenotype of the rice *gd1* mutant. (a) Seed germination of wild-type and *gd1* on MS_0_ for 4 days. (b, c) Seedlings of wild-type and *gd1* after 11 days germination. Scale bars = 2 cm (b) and 1 cm (c). (d) Morphology of wild-type and *gd1* at 2 months after sowing. (e) Panicles of wild-type and *gd1* at the grain-filling stage. (f, g) Iodine staining of pollen grains from wild-type and *gd1*. Five panicles were used for wild-type and mutant plants, and at least 300 pollen grains per panicle were counted. Scale bars = 200 μm.

To identify seed development-related mutants, a large-scale screening was performed using a previously described T–DNA mutant population (Ma *et al*., 2009), and the mutant *germination-defective1* (*gd1*), which is defective in seed germination and seedling development, was obtained. When cultured on MS_0_ medium, wild-type seeds germinate within approximately 2 days and grow normally, but *gd1* germinates and grows much more slowly than wild-type (Figure 1a). At 11 days after germination, the shoots and roots of *gd1* are much shorter than those of wild-type, with approximately one-third of the mutants arrested at the germination stage (Figure 1b,c).

After germination, *gd1* showed distinct dwarf and small leaf phenotypes (Figure 1d). Most of the *gd1* plants died during vegetative growth, and only <10% flowered. The panicle of *gd1* was smaller than that of wild-type, and the flowers also exhibited abnormality (Figure 1e). Rice flowers contain six stamens, one pistil and two stigmas (Figure S1a); however, both the number and identity of the floral organs were altered in *gd1* mutants. *gd1* mutant plants often exhibited seven or eight stamens (Figure S1b,c), and abnormalities were also found in stigmas, with three stigmas often fused together (Figure S1b). In many flowers, an abnormal additional organ appeared (Figure S1d,e), and a stamen-like organ was fused with an imperfect stigma (Figure S1f). Iodine staining showed that approximately 50% of the pollen grains aborted in the *gd1* mutants (Figure 1f,g).

### *GD1* encodes a B3 domain-containing transcription factor

The flanking region of T–DNA was amplified by SiteFinding PCR (Wang. *et al*., 2011), and the amplified fragment was sequenced. BLAST alignment (Altschul *et al*., 1990) revealed that the T–DNA was inserted into the 11th exon of a gene (LOC_Os07 g37610) located on chromosome 7 (Figure S2a,b). RT–PCR analysis showed that the T–DNA insertion led to knockout of full-length gene expression (Figure S2c). However, the gene portions both upstream and downstream of the T–DNA insertion site were still expressed (Figure S2c), as the T–DNA also contains an activation tag (*actin1* promoter) at its left border (Ma *et al*., 2009), which activated expression of the downstream portion of *GD1*. However, we were unable to amplify any products using primers flanking the T–DNA insertion site, indicating that *gd1* is a knockout mutant. *GD1* shares high similarity with the *VAL* genes in Arabidopsis (Figure S2b). The coding sequence of *GD1* contains 2868 bp, which encodes a polypeptide of 956 amino acids. By searching the rice genome (http://rice.plantbiology.msu.edu/), another homolog, *OsVAL2* (LOC_Os07 g48200), was also found. These proteins share five conserved domains: a PHD domain at the *N*–terminus, a B3 domain for DNA binding, a CW-type zinc finger, a nuclear localization signal, and an EAR motif at the *C*–terminus (Figure S2b). It has been shown that EAR motif-containing transcriptional repressors play an important role in modulating plant defense and stress responses (Kazan, 2006). Recently, the PHD domain of HSI2 (HIGH-LEVEL EXPRESSION OF SUGAR INDUCIBLE GENE2)/VAL1 has been implicated in H3K27 trimethylation to repress seed-specific genes in Arabidopsis (Veerappan *et al*., 2012). In addition, a proline- and glutamine-rich extension was found at the *N*–terminus of GD1, which is absent in all other VAL proteins, implying that GD1 may have distinct functions.

**Figure 2:**
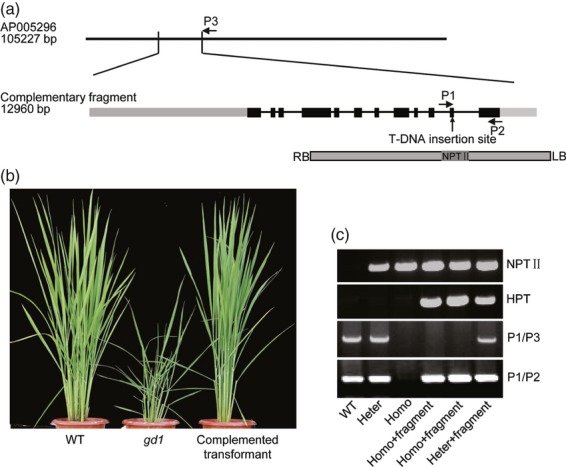
Complementation of the *gd1* mutant. (a) Genomic DNA fragment used for complementation. (b) Complementation restored the *gd1* mutant to a normal wild-type phenotype. (c) Complementation was confirmed by PCR using the primers indicated in (a). Heter, heterozygous *gd1* mutant; Homo, homozygous *gd1* mutant; Homo+fragment, homozygous *gd1* mutant with complementary fragment; Heter+fragment, heterozygous *gd1* mutant with complementary fragment.

To examine whether the T–DNA insertion was responsible for the mutant phenotype, co-segregation analysis between the T–DNA insertion and mutant phenotype in the T_1_ generation was performed. The 666 mutants with a defective phenotype were confirmed to be homozygous for the T–DNA insertion using primers P1, P2 and P4 (Figure S2a), whereas the 692 normal plants were either wild-type (235) or heterozygous (457), indicating that the *gd1* mutant phenotype is due to the T–DNA interruption of *GD1*. For further confirmation, a 13 kb genomic fragment containing the entire 7246 bp *GD1* coding region, a 4614 bp upstream region and a 1092 bp downstream sequence was cloned into binary vector pCAMBIA1300 and transformed into calli derived from the heterozygous seeds (Figure 2a). The individual transformants were confirmed by PCR using primers P1, P2 and P3 shown in Figure 2(a), and transgenic plants with a homozygous *gd1* mutant background were obtained and found to be restored to a wild-type phenotype (Figure 2b,c), showing that loss of function of *GD1* led to the *gd1* mutant phenotype.

### Expression pattern of *GD1*

**Figure 3:**
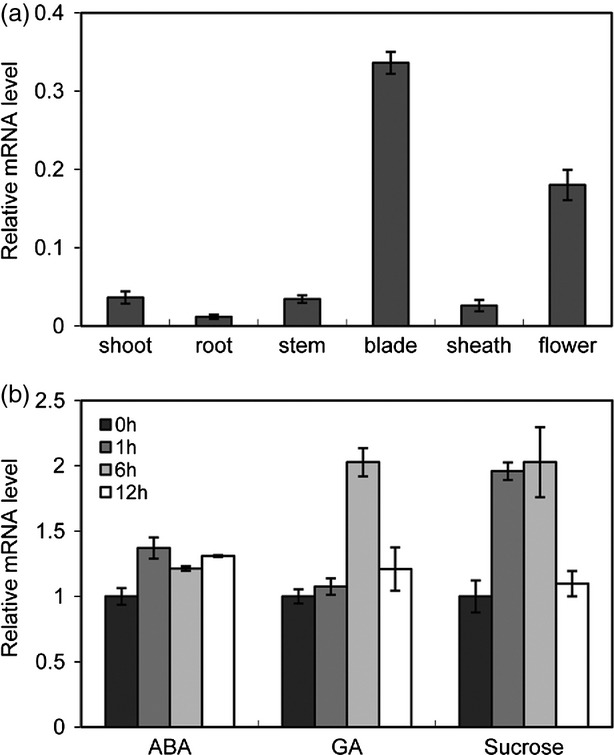
Expression patterns of *GD1*. (a) Quantitative real-time PCR analysis of *GD1* in various organs, including shoot, root, stem, leaf blade, leaf sheath and flower. Values are means ± SD of three biological replicates. (b) Expression levels of *GD1* after treatment with 100 μm ABA, 10 μm GA_3_ and 6% sucrose for various durations. Values are means ± SD of three biological replicates.

Quantitative real-time PCR analysis revealed that *GD1* was ubiquitously expressed in all tissues, including roots, flowers, stems, leaves and calli (Figure 3a). The highest expression level of *GD1* was detected in leaf blades and flowers, whereas the expression level was relatively low in young shoots, leaf sheaths, roots and stems. We next examined the expression patterns of *GD1* under ABA and GA treatments (Figure 3b). The transcript of *GD1* in leaves showed a slight increase upon ABA treatment, but the expression of *GD1* was increased twofold 6 h after GA treatment. We also investigated the effect of sucrose on *GD1* expression, as shown in Figure 3(b), and its expression was strongly up-regulated by sucrose after 1 and 6 h.

### GD1 is a nuclear-localized transcriptional repressor

**Figure 4:**
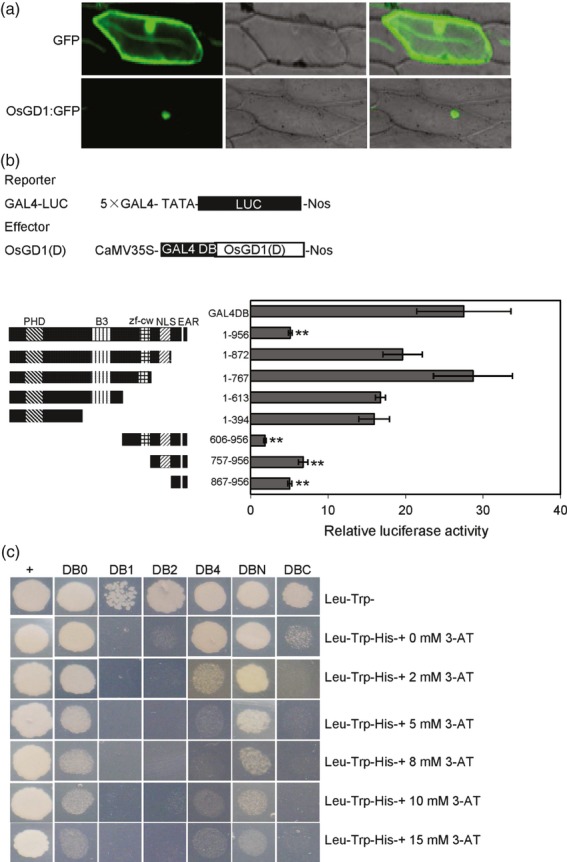
Subcellular localization and repression activity of GD1. (a) Nuclear localization of the GD1–GFP fusion protein in onion epidermal cells. Cells transformed with the control empty plasmid (upper panel) and GD1–GFP plasmid (lower panel) were viewed under a fluorescent filter (left) to show GFP, under a bright field (middle) for cell morphology, and in combination (right). (b) Relative LUC activities after co-transformation of Arabidopsis protoplast with GAL4DB fusion effectors and the GAL4–LUC reporter. Various truncated versions of GD1 were used to construct GAL4DB fusion effectors. The luciferase gene from *Renilla* was used as an internal control. The LUC activity in each assay was normalized relative to that of the internal control. Values are means ± SD. Data are representative of three independent experiments. Student’s *t* test was used for statistical analysis. Asterisks indicate statistically significant differences compared with GAL4DB (**P ≤ 0.01). (c) Transcriptional activity of GD1 in a yeast one-hybrid system. Various truncations of GD1 were constructed as bait vector. DB0, empty control vector; DB1, full-length GD1 (amino acids 1-956); DB2, DB4, DBN and DBC, truncated GD1 constructs (amino acids 1-872, 1-767, 1-481 and 526-956, respectively). All the bait vectors were transformed into yeast stain MaV203 (Invitrogen, Carlsbad, CA, USA). Yeast growth was analyzed at 30°C for 3 days on the SD agar plates lacking Leu, Trp and His and containing various concentrations of 3-amino-1,2,4-triazole (3-AT) as indicated.

To study the subcellular localization of GD1, GD1 was fused with GFP and expressed in onion epidermal cells. As shown in Figure 4(a), fluorescence was detected in both cytoplasm and nucleus for the negative control GFP. In contrast, for the GD1–GFP fusion protein, fluorescence was only detected in the nucleus, suggesting that GD1 is a nuclear-localized protein.

To test whether the EAR motif of GD1 has transcriptional repressor activity, we used a protoplast assay system to detect the transcriptional activity of GD1. In this system, various truncated versions of GD1 were fused with GAL4DB as effector plasmids (Figure 4b), and the firefly luciferase (LUC) gene under the control of five copies of GAL4 was used as the reporter. After introducing both plasmids into protoplasts, the LUC activity was measured to determine the GD1 transcriptional activity. As shown in Figure 4(b), the LUC activity was strongly repressed when the full-length *GD1* effector was co-expressed in the protoplasts. However, the repressed LUC activity was released when various truncated GD1 constructs without the EAR motif (amino acids 1-872, amino acids 1-767, amino acids 1-613 and amino acids 1-394) were co-transformed. Among them, the fragment without the EAR motif and the nuclear localization signal (amino acids 1-767) completely lost its ability to inhibit LUC activity. The LUC activity decreased significantly when various *C*–terminal truncations of the EAR motif (amino acids 606-956, amino acids 757-956 and amino acids 867-956) were used as the effectors (Figure 4b). All these data indicate that the *C*–terminus plays an important role in the repression process, and the EAR motif serves as an active repression domain. Similar results were also obtained from a yeast one-hybrid system (Figure 4c). Both the full-length and *C*–terminal fragment strongly inhibited expression of the reporter gene compared with the empty vector, while transformants containing the *N*–terminal fragment without the EAR motif retained normal gene expression (Figure 4c).

### The B3 domain of GD1 specifically binds to the RY motif

**Figure 5:**
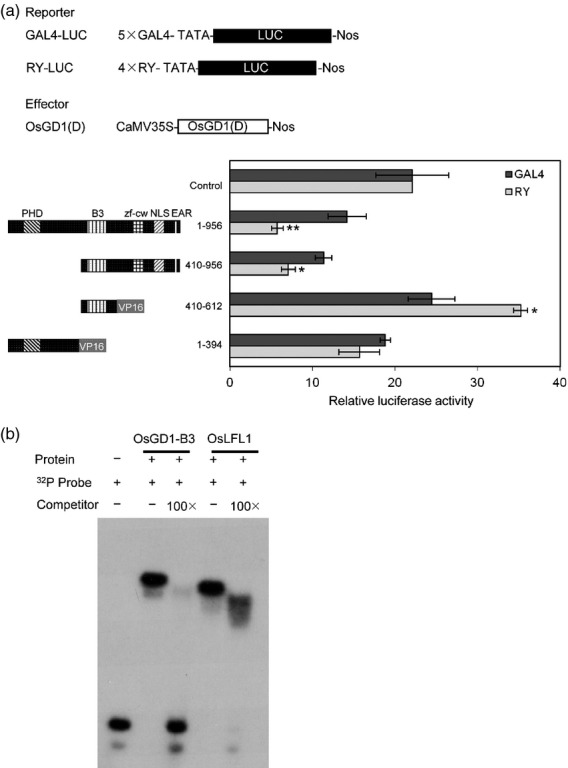
GD1 binds to the RY element. (a) Relative LUC activities after co-transformation of four effectors (amino acids 1-956, the fragment comprising amino acids 410-956 fragment, a fusion of amino acids 410-612 with the VP16 activation domain, and a fusion of amino acids 1-394 with VP16) with the GAL4–LUC and RY–LUC reporters, respectively. Values are means ± SD. Data are representative of three independent experiments. Student’s *t* test was used for statistical analysis. Asterisks indicate statistically significant differences compared with control (*P ≤ 0.05, **P ≤ 0.01). (b) Electrophoretic mobility shift assay indicating that the B3 domain GD1 binds to the RY motif tetramer. *OsLFL1* was used as a positive control. Competition experiments were performed with a 100-fold molar excess of unlabeled competitor DNA oligonucleotides.

Previously it was shown that B3 transcription activators, such as ABI3, FUS3 and LEC2, specifically bind the RY element to positively regulate the expression of downstream target genes (Reidt *et al*., 2000; Monke *et al*., 2004; Braybrook *et al*., 2006). The protoplast expression assay was used to detect whether the B3 domain of GD1 uses a similar binding site. Two LUC reporter plasmids containing 4 × RY DNA-binding elements and 5 × GAL4 binding elements were used (Figure 5a). For the effector plasmids, full-length GD1 (amino acids 1-956) and three truncated versions of GD1 including a *C*–terminal fragment containing the B3 domain (amino acids 410-956), the B3 domain (amino acids 410-612) fused to a VP16 activation domain, and an *N*–terminal fragment (amino acids 1-394) fused to VP16 were constructed (Figure 5a). As shown in Figure 5(a), full-length GD1 and the *C*–terminal fragment containing the B3 domain (amino acids 410-956) greatly inhibited the LUC activity of the RY reporter. When the B3 domain was fused to the VP16 activation domain, the LUC activity of RY reporter was enhanced compared with the GAL4 control. When a fragment without the B3 domain was fused to the VP16 domain, the LUC activity was almost the same in the various reporters. Therefore, the RY motif is a candidate to serve as GD1 binding site, and the B3 domain is responsible for recognition of the target DNA sequence. To further investigate the binding activity of the B3 domain to the RY motif, an *in vitro* gel-shift assay was performed. Due to unsuccessful expression of full-length GD1 in *Escherichia coli*, we used a truncated GD1 containing the B3 domain in the binding experiment. As shown in Figure 5(b), the B3 domain of GD1 directly bound to RY motif tetramer. A competition experiment showed that unlabeled RY oligonucleotide competed for this binding. OsLFL1, which exhibited the same specific binding activity to the RY motif, was used as a positive control. These results suggested that the B3 domain of GD1 bound specifically to the RY motif. Identification of the RY motif as a downstream binding site of GD1 is critical for understanding of the repression mechanism of GD1.

### GD1 negatively regulates GA metabolism

**Figure 6:**
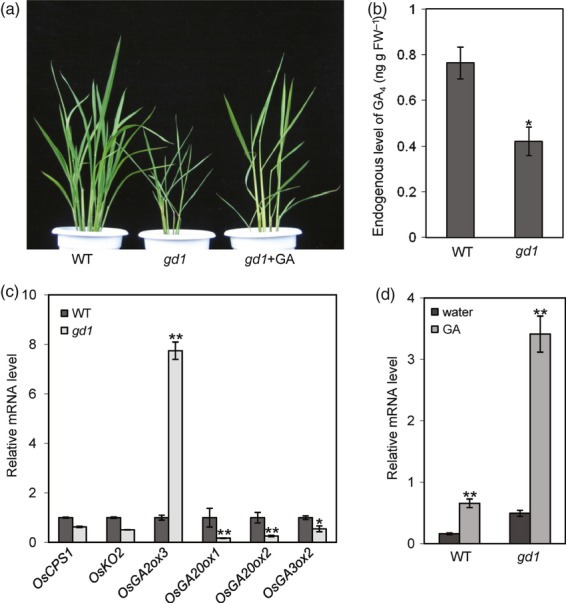
Alteration of GA metabolism in *gd1*. (a) The dwarf phenotype of *gd1* was rescued by GA after spraying with 10 μm GA_3_ for 10 days. Left, wild-type; middle, *gd1*; right, *gd1* sprayed with GA. (b) The endogenous GA_4_ level in *gd1* is lower than in wild-type. Values are means ± SD. Data are representative of three independent experiments. Student’s *t* test was used for statistical analysis. The asterisk indicate a statistically significant difference compared with wild-type (*P ≤ 0.05). (c) Expression patterns of GA synthesis and inactivation genes in 2-month-old wild-type and *gd1* plants by quantitative real-time PCR analysis. Expression levels of each sample were normalized to that of an internal control. Gene expression levels in wild-type were set as 1.0. Values are means ± SD of three biological replicates. Student’s *t* test was used for statistical analysis. Asterisks indicate statistically significant differences compared with wild-type (*P ≤ 0.05, **P ≤ 0.01). (d) Transcripts of *OsGA2ox3* accumulated in *gd1* after exogenous GA treatment for 2 days. Values are means ± SD of three biological replicates. Student’s *t* test was used for statistical analysis. Asterisks indicate statistically significant differences compared with wild-type (**P ≤ 0.01).

B3 transcription factors have been reported to regulate seed germination and seedling development by participating in the ABA and GA pathways. We investigated whether the dwarf phenotype of *gd1* is due to GA deficiency. Four-week-old wild-type and mutant plants were treated with GA_3_, and the response was analyzed 10 days after treatment. As shown in Figure 6(a), GA partially rescued the dwarf phenotype of the *gd1* mutant, indicating that *GD1* may regulate GA homeostasis. In accordance with this result, the endogenous GA_4_ level in the *gd1* mutant was down-regulated compared with wild-type (Figure 6b). Quantitative real-time PCR analysis of several genes involved in GA biosynthesis and catabolism revealed that expression of the GA inactivation gene *OsGA2ox3* was increased dramatically, while expression of GA biosynthetic genes, including *OsGA20ox1*, *OsGA20ox2* and *OsGA3ox2*, was decreased in the *gd1* mutant (Figure 6c and Figure S3). In addition, *OsCPS1* and *OsKO1* were found to be down-regulated in the leaves of 2-month-old *gd1* mutant plants (Figure 6c). Interestingly, *OsCPS1* and *OsKO1* were significantly up-regulated in the leaves of 4-month-old mutant plants (Figure S3). Moreover, expression of *OsGA2ox3* was significantly induced in *gd1* after exogenous GA treatment for 2 days (Figure 6d). These results indicate that *GD1* is involved in the regulation of GA metabolism, either directly or more likely through other components.

### Expression of *OsLFL1* is upregulated in the *gd1* mutant

**Figure 7:**
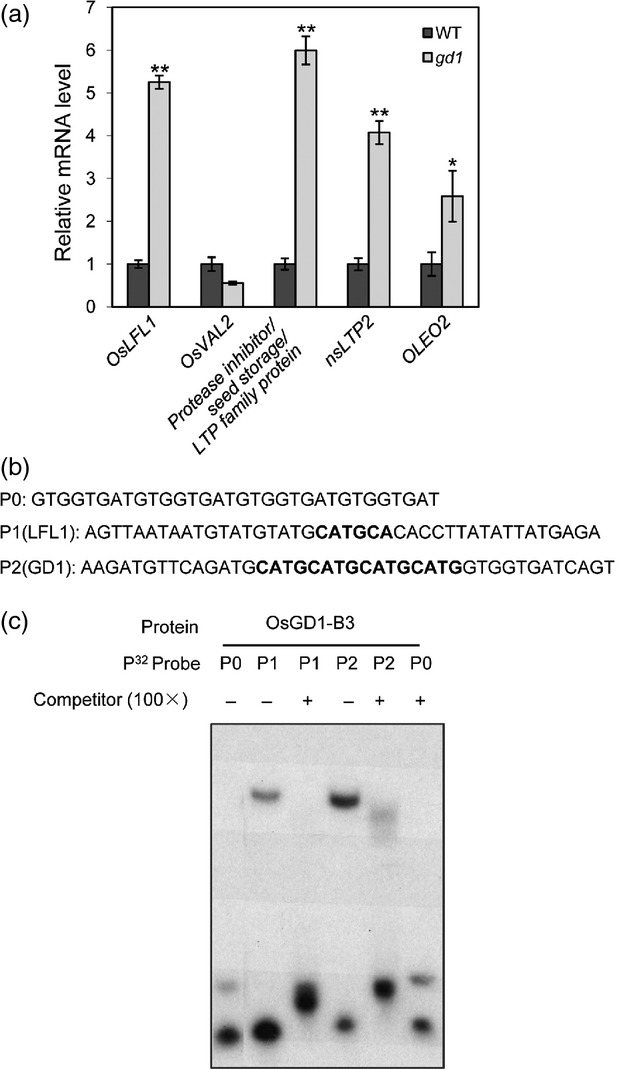
GD1 protein can bind directly to the RY motif in the *OsLFL1* promoter. (a) Quantitative real-time PCR analysis of *OsLFL1*, *OsVAL2* and other genes including that encoding protease inhibitor/seed storage/LTP family protein, *non-specific* *LTP2* and *OLEO2* in 2-month-old wild-type and the *gd1* mutant. *OsActin1* was used as an internal standard. Expression levels of each gene in *gd1* were normalized to that of wild-type internal control. Values are means ± SD of three biological replicates. Student’s *t* test was used for statistical analysis. Asterisks indicate statistically significant differences compared with wild-type (*P ≤ 0.05, **P ≤ 0.01). (b) Sequences of DNA probes derived from the promoters of *OsLFL1* and *GD1*. P0 does not contain a RY element. The RY elements are indicated in bold. (c) Electrophoretic mobility shift assays with the recombinant B3 domain of GD1 in the presence and absence of the indicated unlabeled competitor DNA oligonucleotides. Competition experiments were performed with a 100-fold molar excess of competitor.

It has been reported that expression of *VAL* genes in Arabidopsis represses the expression of *LEC1* and the B3 network in developing seeds, which is essential for repression of embryonic pathways (Suzuki *et al*., 2007; Suzuki and McCarty, 2008). Due to the defect in seed germination, we speculate that *GD1* may be involved in transition between the seed and vegetative phases. We first compared the expression level of *OsLFL1* in *gd1* mutant and the wild-type. Quantitative real-time PCR results showed that *gd1* had a higher level of the *OsLFL1* transcript (Figure 7a). OsLFL1 is the only B3 domain-containing protein characterized in rice so far, and probably has similar functions to LEC2 and FUS3 (Peng *et al*., 2007, 2008). The increased expression of *OsLFL1* in *gd1* suggested that the negative effect of *GD1* on the embryonic pathway may be similar to the results found in Arabidopsis. Furthermore, we identified RY elements within the 1.5 kb promoter regions of *OsLFL1* and *GD1*. As expected, this DNA-binding study provided additional evidence that the B3 domain of GD1 is directly involved in regulation of *OsLFL1* and *GD1* itself (Figure 7b,c). In addition, genes including one encoding a protease inhibitor/seed storage/LTP family protein, non-specific *LTP2* and *OLEO2* were also expressed at higher levels in the *gd1* mutant compared to wild-type (Figure 7a). Although we do not know their exact roles in seed development, the accumulation patterns suggest that they are downstream targets of B3 proteins. During germination, failure to repress *OsLFL1* and many seed development-associated genes is probably responsible for the developmental retardation phenotypes in *gd1*. Interestingly, expression of *OsVAL2* was found to be down-regulated in *gd1* (Figure 7a).

### Plants over-expressing *OsLFL1* partly phenocopy the *gd1* mutant

**Figure 8:**
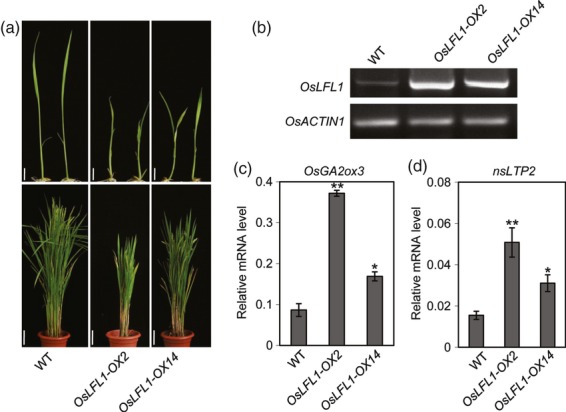
Over-expression of *OsLFL1* partly phenocopies *gd1* mutant. (a) Phenotypes of *OsLFL1* over-expression lines. Top: 2-week-old stage; scale bars = 2 cm. Bottom: grain-filling stage; scale bars = 10 cm. (b) RT–PCR confirmation of over-expression of *OsLFL1* in the two *OsLFL1* over-expression lines. (c) Determination of the transcript level of *OsGA2ox3* and *nsLTP2* in *OsLFL1* transgenic lines by quantitative real-time PCR. The data were referenced to *OsActin1* as an internal control and normalized against wild-type. Values are means ± SD of three biological replicates. Student’s *t* test was used for statistical analysis. Asterisks indicate statistically significant differences compared with wild-type (*P ≤ 0.05, **P ≤ 0.01).

As very few homozygous *gd1* seeds were obtained, making it difficult to generate *OsLFL1* knockdown transgenic plants in the *gd1* background, *OsLFL1* over-expression lines were generated to further determine the role of *OsLFL1* in mediating the *GD1-*regulated GA homeostasis. *OsLFL1* transgenic lines showed a similar dwarf phenotype to *gd1* during seedling and adult plant growth periods (Figure 8a). In addition, ectopic expression of *OsLFL1* in rice also causes dwarf stature and small leaves, which resemble the *gd1* phenotypes except for the delay in flowering. We used the *OsLFL1–2* and *OsLFL1–14* lines for further expression analysis (Figure 8b). As expected, expression of *OsGA2ox3* and non-specific *LTP2* was induced in *OsLFL1* over-expression plants (Figure 8c), mimicking the expression patterns in the *gd1* mutant (Figures 6c and 7a). These results suggest that *OsLFL1* is the downstream target of *GD1*.

### Carbohydrate metabolism is affected in the *gd1* mutant

**Figure 9:**
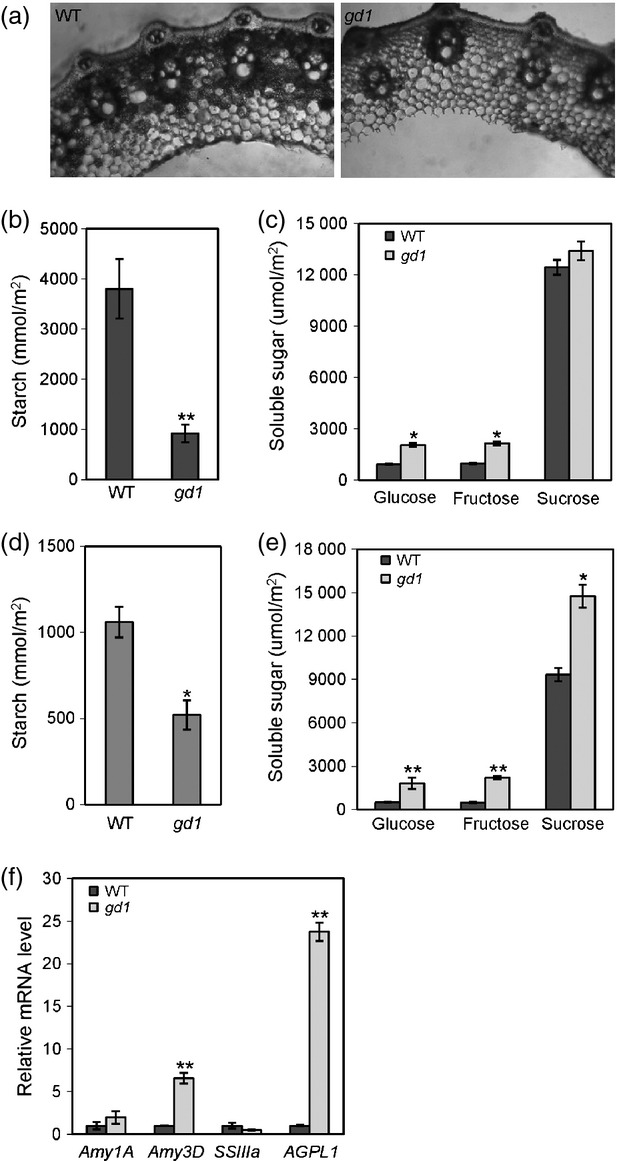
Regulation of carbohydrate metabolism in the *gd1* mutant. (a) Reduced number of starch granules in cross-sections of stems of wild-type and *gd1*. (b, c) Content of starch and soluble sugars including glucose, fructose and sucrose in the flag leaves of wild-type and *gd1*. Values are means ± SE (*n* = 10). Similar results were obtained for three independent experiments. Asterisks indicate statistically significant differences compared with wild-type (*P ≤ 0.05, **P ≤ 0.01). (d, e) Content of starch and soluble sugars including glucose, fructose and sucrose in the top second leaves of wild-type and *gd1*. Values are means ± SE (*n* = 10). Similar results were obtained for three independent experiments. Asterisks indicate statistically significant differences compared with wild-type (*P ≤ 0.05, **P ≤ 0.01). (f) Expression pattern of carbohydrate metabolism-associated genes in wild-type and *gd1*. Values are means ± SD of three biological replicates. Student’s *t* test was used for statistical analysis. Asterisks indicate statistically significant differences compared with wild-type (**P ≤ 0.01).

In Arabidopsis, the *val1* mutant showed altered expression of sugar response genes. Interestingly, we found that the number of starch granules was significantly reduced in the stem of the *gd1* mutant compared to the wild-type (Figure 9a). Measurement of the carbohydrate content in the leaves also revealed that the starch content decreased significantly in both flag leaves and the top second leaves of the *gd1* mutant (Figure 9b,d); however, soluble sugars including glucose, fructose and sucrose accumulated to high levels in *gd1* (Figure 9c,e), suggesting that GD1 participates in regulation of carbohydrate metabolism. To study the potential involvement of GD1 in carbohydrate metabolism, the expression patterns of some related genes were further investigated. As shown in Figure 9(f), expression of the α–amylase genes *RAmy1A* and *RAmy3D* was induced, whereas expression of the transcript of *SSIIIa* (encoding starch synthase) was inhibited in the *gd1* mutant (Figure 9f), consistent with the carbohydrate alteration observed in the mutants. One exception is ADP-glucose pyrophosphorylase large subunit 1 (encoded by *AGPL1*), which catalyzes a rate-limiting step of starch biosynthesis in plants. AGPase family genes are known to accumulate in response to sugars and ABA (Akihiro *et al*., 2005; Ohdan *et al*., 2005). Expression of *AGPL1* was elevated in the *gd1* mutant, which may be explained via feedback regulation by the increased endogenous sugar content.

## Discussion

Seed development and germination are highly complex and fine-tuned developmental processes (Finkelstein *et al*., 2008; Santos-Mendoza *et al*., 2008). During recent years, huge amounts of work have been performed in Arabidopsis and maize (*Zea mays*) to identify components associated with seed maturation and germination. Many of these are involved in GA and ABA signaling, biosynthesis or catabolism (White *et al*., 2000; Finkelstein *et al*., 2002; Olszewski *et al*., 2002; Peng and Harberd, 2002; Nambara and Marion-Poll, 2003, 2005; Gubler *et al*., 2005; Fang *et al*., 2008; Suzuki *et al*., 2008; Nakashima *et al*., 2009). The key roles played by ABA and GA in regulating seed development and germination are well-established (Piskurewicz *et al*., 2008, 2009), and reduction of ABA or increase of GA in seeds results in a switch from the seed maturation program to the germination program (Koornneef *et al*., 2002; Finch-Savage and Leubner-Metzger, 2006). In addition, roles of sugar and other hormones in regulation of seed germination have also been proposed (Gibson, 2004; Rolland *et al*., 2006; Yuan and Wysocka-Diller, 2006); however, the molecular mechanism is still poorly understood. Recent studies indicate that cross-talk between ABA- and auxin-dependent responses occurs during seed germination and early seedling development in which ABA-dependent repression of growth is potentiated by auxin (Belin *et al*., 2009).

Rice is one of the most important crops in the world. The correct transition from seed maturation to germination is an important agronomic trait. Although several loci have been identified that regulate rice seed maturation and germination processes, the complete pathway and the underlying mechanism remain unclear (Agrawal *et al*., 2001; Fang *et al*., 2008). Here, we identified a *gd1* mutant from a T–DNA mutant population that exhibits defect in seed germination and seedling development, including dwarf and sterile phenotypes. *GD1* is expressed ubiquitously and is regulated by hormone and sugar treatment, indicating an essential role in plant development.

*GD1* encodes a B3 domain-containing protein that shares high similarity with VAL proteins in Arabidopsis. Previous reports in Arabidopsis have shown that two sub-families of B3 domain transcription factors, including ABI3/FUS3/LEC2 and VAL, are critical determinants in seed and vegetative development (Suzuki and McCarty, 2008). AtVAL1 functions as a transcriptional repressor for sugar-inducible genes and plays an important role during the transition from seed maturation to germination (Tsukagoshi *et al*., 2005, 2007; Suzuki *et al*., 2007). The VAL family in Arabidopsis is required for repression of the LEC1/B3 transcription factor network in germinating seedlings (Suzuki *et al*., 2007). Except for the B3 domain, VAL sub-family proteins do not have any other similarities to ABI3/FUS3/LEC2 proteins. AtVAL1 and related proteins have been reported to be B3 domain–EAR motif active transcription repressors (Tsukagoshi *et al*., 2005). A number of reports have indicated that EAR motif-containing proteins play a key role in the transcriptional repression of plant defense- and stress-associated gene expression (Ohta *et al*., 2001; Sakamoto *et al*., 2004; Song *et al*., 2005; Yang *et al*., 2005; Kazan, 2006; Kagale and Rozwadowski, 2010). Our transient expression analysis in both Arabidopsis protoplasts and yeast indicated that the EAR motif in the *C*–terminus of GD1 functions as an active repression domain, suggesting that GD1 is involved in seed germination and seedling development by negatively regulating target gene expression.

Currently, our knowledge about the function of B3 genes in grass species is still poor and fragmentary. In rice, there are several proteins that are homologous to the products of the Arabidopsis genes, but only one of them, OsLFL1, which is homologous to FUS3, has been studied in depth (Peng *et al*., 2007). Expression of *OsLFL1* was enhanced in the *gd1* mutant, consistent with the results in Arabidopsis. In combination with the evidence in Arabidopsis, which demonstrates the positive regulatory role of the *ABI3/FUS3/LEC2* genes in the embryonic pathway, de-repression of *OsLFL1* in the *gd1* mutant partially explains *gd1* defective phenotype in germination. These results suggest that GD1 has a similar function to AtVAL proteins. The molecular mechanisms controlling the embryo pathway are probably conserved between monocot and dicot species, at least to some extent. Like other B3 factors, GD1 binds to the RY element through the B3 domain. Interestingly, more than 10 RY elements were found within the 1.5 kb genomic sequence upstream of *GD1*. In addition, one RY element was also found in the upstream sequence of *OsLFL1*. Gel-shift experiments revealed that GD1 binds to the promoters of both *GD1* and *OsLFL1*, indicating that GD1 regulates the expression of itself and other B3 factors. However, in *OsLFL1* over-expression lines, the transcript levels of *GD1* and its homolog remain the same as in wild-type. All the data above suggest that *OsLFL1* functions downstream of GD1. The data presented also demonstrate the complex regulation network within B3 transcription activators and repressors. In order to further elaborate how GD1 regulates seed germination and seedling development, several ChIP experiments for testing the interaction between GD1 and OsLFL1 *in vivo* were performed, but the unstable nature of the GD1 protein made all our attempts failed.

**Figure 10:**
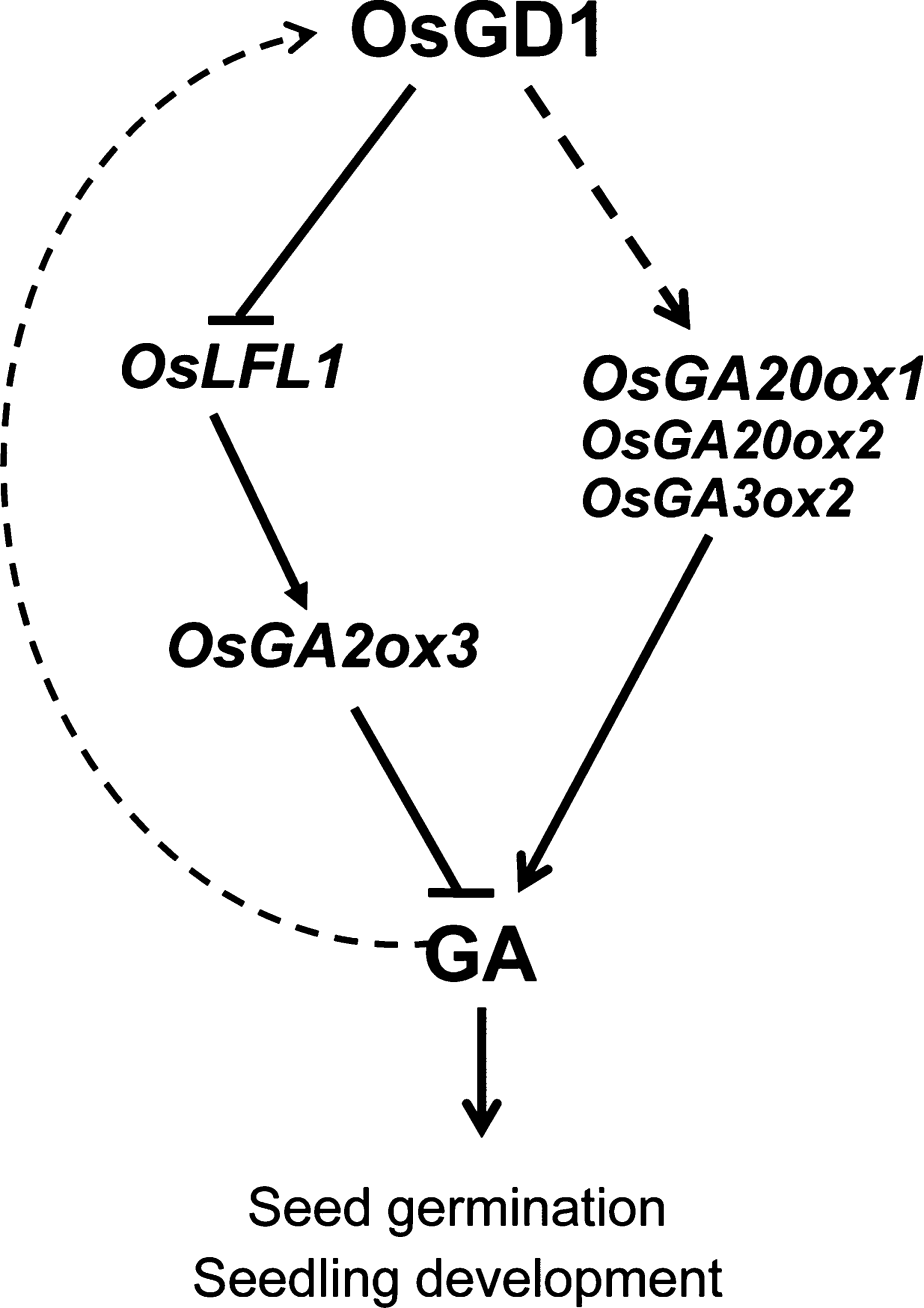
Proposed model for GD1-regulated GA homeostasis.

The phenotypes of most adult *gd1* mutants are reminiscent of plants that are defective in GA synthesis or action. Expression of *GD1* may be induced by GA, the endogenous GA level was significantly reduced in *gd1*, and the dwarf phenotype of the *gd1* mutant may be partially rescued by exogenous application of GA. Expression analysis clearly showed that expression of the GA deactivation gene *OsGA2ox3* was strongly induced in *gd1* under GA treatment compared with wild-type; however, the expression levels of the GA biosynthesis genes *OsGA20ox1*, *OsGA20ox2* and *OsGA3ox2*, were decreased in the *gd1* mutant. These data suggest that GD1 participates in maintaining GA homeostasis by positively regulating GA biosynthesis and negatively regulating GA deactivation. Inconsistent expression changes for *OsCPS1* and *OsKO1*, which are also involved in GA biosynthesis, were probably due to the differentially developmental manner (Silverstone *et al*., 1997; Smith *et al*., 1998; Hedden and Phillips, 2000). The oscillation of GA concentration is critical for regulating seed germination and seedling development. The levels of bioactive GAs are maintained via feedback and feed-forward regulation of GA metabolism, and several factors that influence GA metabolism have been identified (Yamaguchi, 2008). Microarray data analysis in *atval* mutants suggested that *VAL* genes may be involved in the regulation of GA synthesis, because *AtGA3ox1*, one of the key GA biosynthesis genes expressed during seed germination, is down-regulated more than 10-fold in the *val1/val2* double mutant compared to wild-type (Suzuki *et al*., 2007). GA biosynthesis was also shown to be regulated by the LEC2 and FUS3 pathways (Curaba *et al*., 2004; Gazzarrini *et al*., 2004). Lines over-expressing *OsLFL1* showed dwarf stature, small leaves and leaf-like petals, and GA metabolism genes showed a similar expression pattern to the *gd1* mutant (Peng *et al*., 2007). Therefore, we can derive a scenario for the possible role of *GD1* in the regulation of B3 transcription factors and GA metabolism: GA induces *GD1* expression, and GD1 negatively regulates B3 transcription factors such as *OsLFL1* and *GD1* itself, then directly or indirectly modulates the GA level, regulating seed germination and seedling development (Figure 10).

Sugars, as energy sources and structural components, have important functions in plant development. Many genetic approaches have been used to identify the regulatory pathways that control sugar signaling in Arabidopsis. Various alleles of *aba1*, *aba2*, *aba3*, *abi4* and *abi5* were isolated that have altered sugar responses, indicating a close correlation between the sugar and ABA pathways (Fedoroff, 2002; Rook *et al*., 2006). Consistent with the results obtained for *VAL1*, *GD1* showed increased expression under sugar treatment. The contents of starch and soluble sugars are altered in *gd1*, suggesting that GD1 is involved in regulation of the metabolism of starch and sugar. The expression of genes involved in carbohydrate metabolism also varied between *gd1* and wild-type. The enhanced expression of *AGPL1* is indicative of a high endogenous sugar level and ABA level. However, the molecular basis of how GD1 is involved in the carbohydrate metabolism has not yet been determined.

In conclusion, GD1 plays a critical role in the regulation of B3 transcription factors, and also contributes to GA and carbohydrate catabolism regulation. Correct expression of *GD1* is crucial for proper seed and plant development. To better understand the regulatory role of GD1, future work will focus on identification of its interaction partners and downstream targets.

## Experimental Procedures

### Plant materials and growth conditions

All rice lines used in this study were derived from the *japonica* cultivar Nipponbare. The plants were grown in paddy fields under natural conditions except where specifically indicated. Germination of *gd1* was assessed on plates containing MS_0_ medium (Sripaoraya *et al.,* 2003), using seeds harvested from heterozygous *gd1* plants.

For various treatments, the rice seeds were pre-soaked for imbibition in water at 37°C for 2 days. Then the soaked seeds were sown and cultivated in a growth chamber at 28°C with constant light. Two-week-old seedlings were subjected to treatment with 100 μm ABA, 10 μm GA_3_ or 6% sucrose, respectively. The aerial parts of treated or control seedlings were harvested at the 0, 1, 6 and 12 h time points. For the rescue of the *gd1* phenotype by GA plants grown in the soil for 2 weeks were sprayed with 10 μm GA_3_ for 10 days. For gene expression analysis, seedlings were sampled after spraying 10 μm GA_3_ treatment for 2 days.

### Vector construction and rice transformation

A 13 kb genomic DNA fragment containing the 7246 bp entire *GD1* coding region, a 4614 bp upstream region and a 1092 bp downstream sequence was inserted into the binary vector pCAMBIA1300 (CAMBIA, Canberra, Australia) by digesting with *Xho*I and *Sal*I to generate a transformation plasmid for the complementation test. The plasmid was introduced into *Agrobacterium tumefaciens* AGL1 by electroporation, and rice transformation was performed as previously described (Liu *et al*., 2007). Calli derived from seeds of heterozygous plants were used. The genotype of each transformant was determined by PCR. For PCR, the P1 (5′-GAGCTTGGCGAAATGATTCCTA-3′), P2 (5′-CTAGGTTGGGTTGTCCCTGAGG-3′), and P3 (5′-GGCACATGTTCTGTTCTTCTGTGTT-3′) primers were used.

To generate the *OsLFL1* over-expression vector, the full-length cDNA fragment of *OsLFL1* was amplified using primers5′-CCCGCAGGGAATCCCGATG-3′ and 5′-CGCCAGGGAAGGGGTTTCTG-3′. Then the fragment was cut using *Xba*I and *Sal*I, and inserted into the pCAMBIA2300 (CAMBIA, Canberra, Australia) vector.

### Identification of the T–DNA insertion site

The flanking sequence of the inserted T–DNA fragment in the mutant was determined using SiteFinding PCR as described previously (Wang. *et al*., 2011). The products of PCR were cloned into the pGEM-T-easy (Promega, Madison, WI, USA) and then sequenced. Genotyping of the segregating population was performed by PCR using P1, P2, and P4 (5′-CGTCCGCAATGTGTTATTAAGTTGTCT-3′).

### RT–PCR analysis for the *gd1* transcript

A total of 200 ng RNA was used for RT–PCR reactions (30 cycles) in a total volume of 20 μL using an RT–PCR kit (Toyobo, Osaka, Japan). The primers used for RT–PCR were P5 (5′-GCCTGCCAATGGTGTACTACCC-3′), P6 (5′-GTCCTCCTCCCAAGTATTGGTGG-3′), P7 (5′-TCTGTATGACAGTGCGACGCC-3′), *OsActin1F* (5′-TGAGACCTTCAACACCCCTG-3′) and *OsActin1R* (5′-TCTTGGCAGTCTCCATTTCC-3′).

### RNA preparation and real-time quantitative PCR

Total RNAs were isolated using the guanidinium isocyanate/acidic phenol method (Chomczynski and Sacchi, 1987). Synthesis of first-strand cDNA and real-time PCR analysis were performed as described previously (Yang *et al*., 2009). *OsActin1* was used as an internal control. Gene-specific primers were designed and are listed in Table S1. Three biological repeats were performed for each gene.

### Subcellular localization of GD1–GFP fusion proteins

The *GD1* cDNA was amplified using primers 5′-CTTAGGGTTTCGAGGCCCA-3′ and 5′-GAGCTCCTAGGTTGGGTTGTCCCTGAGG-3′, and cloned into the *Nhe*I and *Sac*I sites of the transient expression vector pGFP2(GA)5II. Transient expression assays of GFP localization in onion epidermal cells were performed by particle bombardment (model PDS–1000, Bio–Rad, Hercules, CA, USA). After overnight incubation in the dark, confocal images were collected using a Zeiss LSM 510 Meta confocal laser scanning microscope (Jena, Germany).

### Transient expression assay in protoplasts and LUC activity determination

For the transcriptional repression activity assay, the reporter plasmid GAL4–LUC and effector plasmids with various lengths of GD1 were used. The GAL4–LUC plasmid includes five repeats of the yeast GAL4 protein binding site, and the minimal TATA region of the CaMV 35S promoter located upstream of the firefly luciferase gene (Fujimoto *et al*., 2000; Ohta *et al*., 2000). The effector plasmids were constructed by fusing various lengths of the GD1 coding region (amino acids 1-956, 1-872, 1-767, 1-613, 1-394, 606-956, 757-956 and 867-956) with GAL4DB. Arabidopsis mesophyll protoplasts were isolated and transformed by the poly(ethylene glycol)-mediated method (Yoo *et al*., 2007). To normalize values, plasmid pPTRL (Ohta *et al*., 2001), which includes a luciferase gene from *Renilla* reniformis under the control of the CaMV 35S promoter, was used as an internal control. Luciferase activity was quantified using a dual-luciferase reporter assay kit (Promega, Madison, WI, USA). The results are reported as the ratio of firefly relative light units (RLUs) versus *Renilla* RLUs as obtained using a luminometer (Promega). Experiments were performed in triplicate.

In order to explore the relationship between GD1 and the RY element, a reporter plasmid with 4 × RY DNA-binding element (CATGCATGCACATGCATGTGCATGCATGCGCATGCATGAG) was constructed. Four effectors (amino acids 1-956, the fragment comprising amino acids 410-956 fragment, a fusion of amino acids 410-612 with the VP16 activation domain, and a fusion of amino acids 1-394 with VP16) were co-transformed with GAL4–LUC and RY–LUC reporters, respectively. The RY–LUC plasmid includes four repeats of RY motif. Luciferase activity was then assayed.

### Electrophoretic mobility shift assays

Gel-shift assays were performed as described by the manufacturer of the equipment used (Promega). Because of failure to obtain full-length GD1 protein after many attempts, we prepared only the B3 domain of GD1 protein containing amino acids 387–613 using *E. coli* strain BL21 (DE3). *OsLFL1* was used as a positive control. For *GD1*, the oligonucleotides 5′-GAATTCGCTTCCTCCTCATTGCAGAA-3′ and 5′-TCGTCGACACAGGCAGTGGCTTGTTCCAC-3′ were used, and for *OsLFL1* the oligonucleotides 5′-CCGGATCCGCCCGTCCCACCGACATG-3′ and 5′-AAGTCGACCAGGGAAGGGGTTTCTGAGCTTC-3′ were used. Fragments were sub-cloned into expression vector pGEX6P–1 (Amersham Pharmacia Biotech, Tokyo, Japan). The glutathione S-transferases (GST) fusion protein was extracted from bacteria utilizing glutathione-tagged Sepharose (GE Healthcare, Piscataway, NJ, USA). The eluted GST fusion protein was assayed by SDS–PAGE.

To test whether GD1 bind RY elements, complementary 59 bp single-stranded oligonucleotides containing the RY motif tetramer TGCATGCATGCATGCATGGTGCCATGCATGCAGGTGCATGCAGACGCATGCATGCACC were synthesized. For downstream gene promoter binding experiments, complementary single-stranded oligonucleotides derived from their promoters were synthesized as DNA probes. To obtain double-stranded RY motif-containing fragments, two complementary oligonucleotides were mixed and heated in a water bath at 95°C for 5 min, and cooled to room temperature for annealing.

### GA_4_ quantification

Quantification of endogenous GAs was performed as described previously (Li *et al*., 2011). Rice panicles (approximately 3 g fresh weight) of *gd1* and wild-type were harvested 100 days after sowing.

### Carbohydrate analysis

Carbohydrate analysis was performed as described previously (Stitt *et al*., 1989). For extraction of soluble sugars and starch, two leaf discs (9 mm diameter) were taken from the fully expanded flag leaves or top second leaves of field-grown plants using a cork borer, and frozen immediately in liquid nitrogen.

## Supporting Information

Figure S1 Morphology of flowers in wild-type and the *gd1* mutant.

Figure S2 Cloning of the *GD1* gene.

Figure S3 Expression analysis of GA synthesis genes and inactivation genes in 4-month-old wild-type and *gd1* plants by quantitative real-time PCR.

Table S1 Primers used for quantitative real-time PCR.
