# Supplementary figures and images for: The rice GERMINATION DEFECTIVE 1, encoding a B3 domain transcriptional repressor, regulates seed germination and seedling development by integrating GA and carbohydrate metabolism
Source: Plant J. 2013 May 13;75(3):403–16. doi: 10.1111/tpj.12209 (PMC3813988; doi:10.1111/tpj.12209)

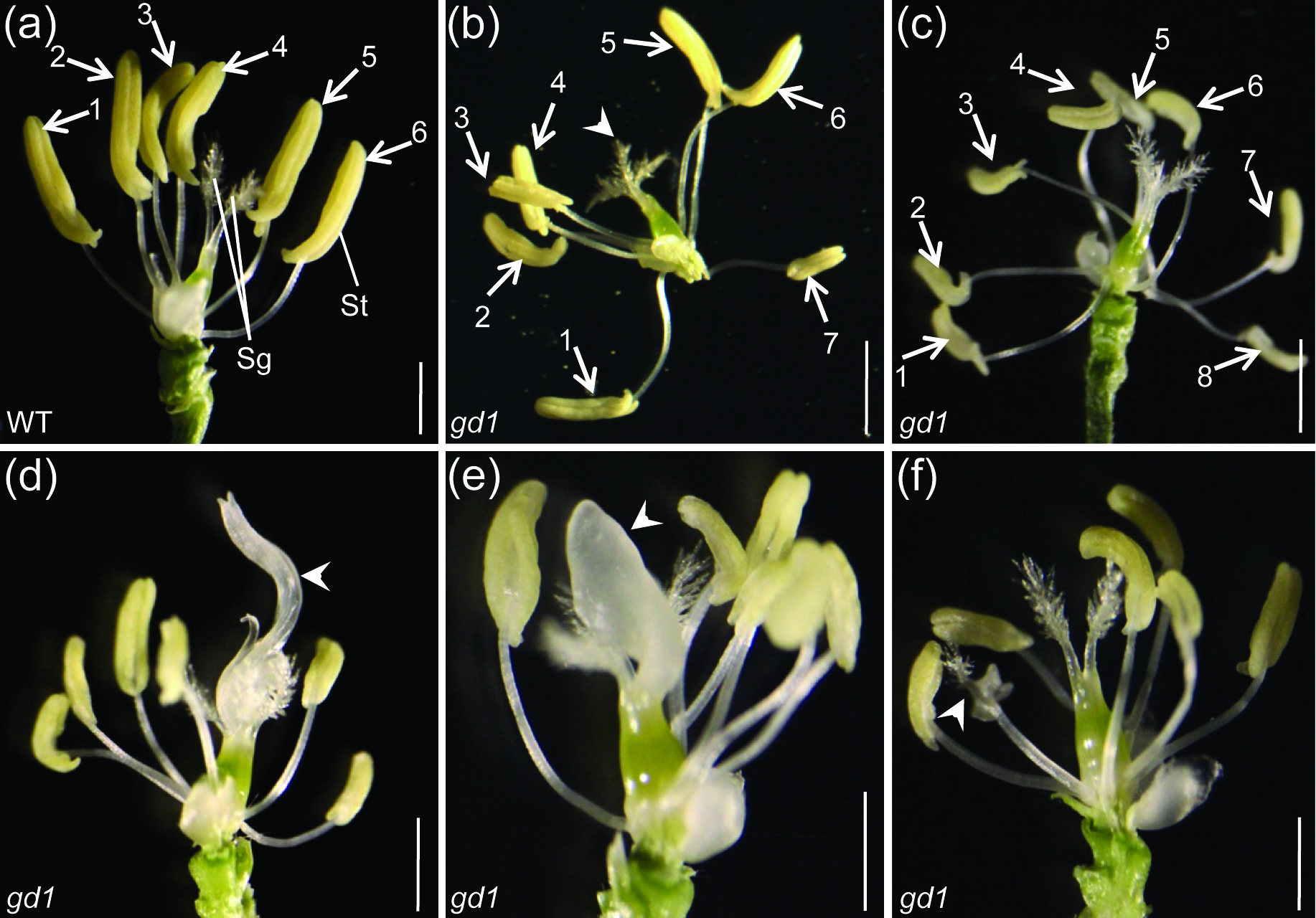

Supplement: Figure S1 — Morphology of flowers in wild-type and the gd1 mutant. [file tpj0075-0403-sd1.tif]

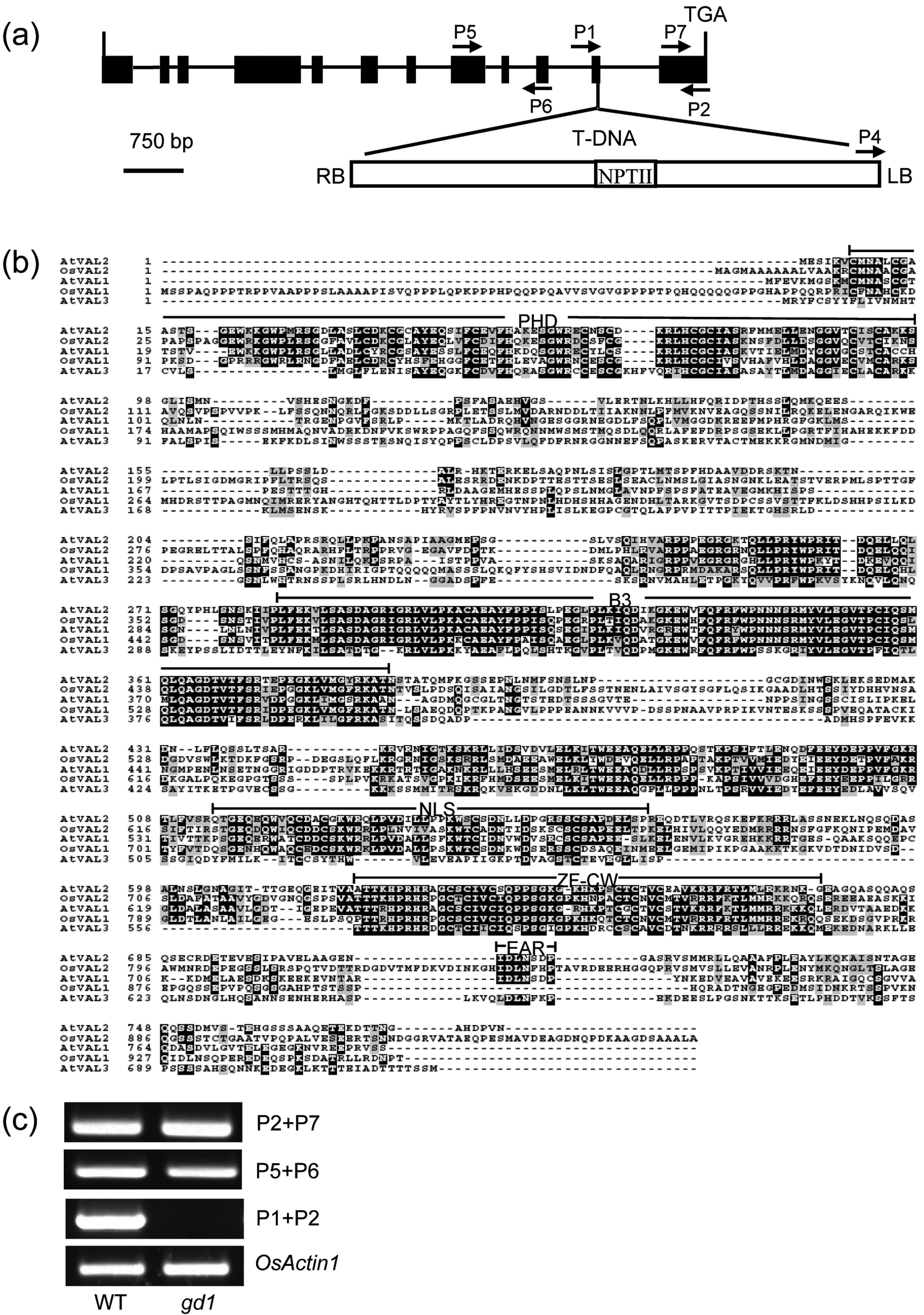

Supplement: Figure S2 — Cloning of the GD1 gene. [file tpj0075-0403-sd2.tif]

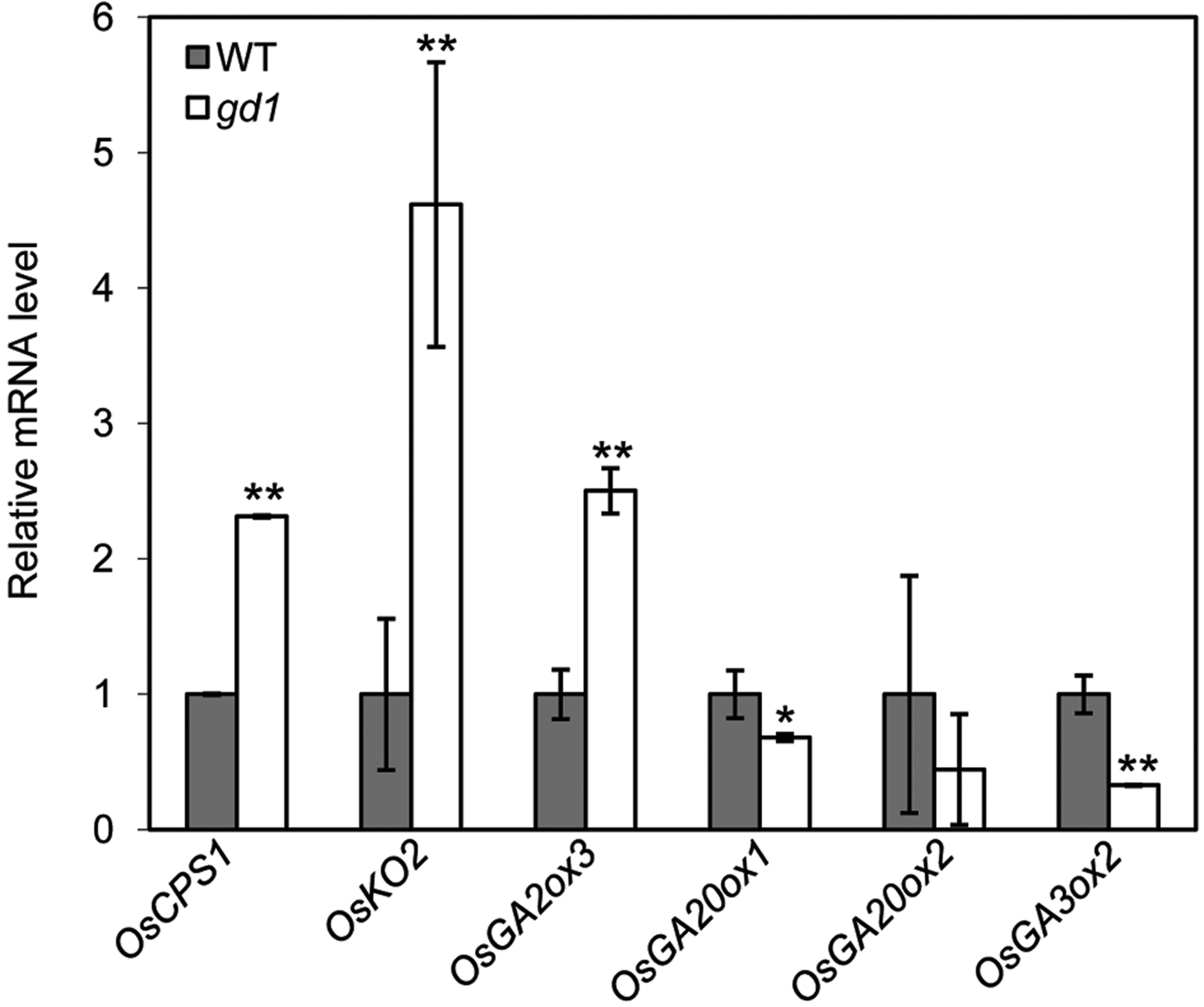

Supplement: Figure S3 — Expression analysis of GA synthesis genes and inactivation genes in 4-month-old wild-type and gd1 plants by quantitative real-time PCR. [file tpj0075-0403-sd3.tif]
