# Supplementary material for: The rice GERMINATION DEFECTIVE 1, encoding a B3 domain transcriptional repressor, regulates seed germination and seedling development by integrating GA and carbohydrate metabolism
Source: Plant J. 2013 May 13;75(3):403–16. doi: 10.1111/tpj.12209 (PMC3813988; doi:10.1111/tpj.12209)
Supplement: Table S1 — Primers used for quantitative real-time PCR. [file tpj0075-0403-sd4.docx]

| **Gene** | **Primer sequences** |
| --- | --- |
| *GD1* | 5'GCCTGCCAATGGTGTACTACCC'3 |
|  | 5'GTCCTCCTCCCAAGTATTGGTGG'3 |
| *OsLFL1* | 5'GCAAAATGCACAACTCTGGACC'3 |
|  | 5'GGCACAGTGGCAGCTTGTTG'3 |
| *OsVAL2* | 5'TGTTCCTGCTCTGCACCTGAAG'3 |
|  | 5'CGACATCACCATAGACTGCGGC'3 |
| *Protease inhibitor/*  *seed storage/LTP family protein* | 5'CGCCTCCGAACACGCAGT'3 |
|  | 5'TGGCGGCGACGTTGTTG'3 |
| *nsLTP2* | 5'CGGAGGGTGTGACATCGCA'3 |
|  | 5'GCTAGTCTCGTCATGCATGGCG'3 |
| *OLEO2* | 5'GGTTTCGCTTCGCTTCGTAGTAG'3 |
|  | 5'CCGACCTCGCTTATTATCATTCAC'3 |
| *OsCPS1* | 5'GTGCTGGACGAATTGAGGAGG'3 |
|  | 5'GCATACCCAACTCAATTTCCTTG'3 |
| *OsKO2* | 5'CGGAGTCCATCCTGGCTGC'3 |
|  | 5'CGGCGACTCCCACACCTTC'3 |
| *OsGA2ox3* | 5'CAGAGGATTGCACCATTGCCAC'3 |
|  | 5'GTCGTTGTTGCTTGACCGGC'3 |
| *OsGA20ox1* | 5'AATGAGCATGGTGGTGCAGCAGGAGCAG'3 |
|  | 5'GTTAACCACCAGGAAGAAGCCGTGCCTC'3 |
| *OsGA20ox2* | 5'TACTACAGGGAGTTCTTCGCGGACAGCA'3 |
|  | 5'TGTGCAGGCAGCTCTTATACCTCCCGTT'3 |
| *OsGA3ox2* | 5'TCTCCAAGCTCATGTGGTCCGAGGGCTA'3 |
|  | 5'TGGAGCACGAAGGTGAAGAAGCCCGAGT'3 |
| *AGPL1* | 5'ATGCAGTGCAGTGCGTCTTT'3 |
|  | 5'ACTTCACTCGGGGCAGCTTA'3 |
| *SSIIIa* | 5'GCCTGCCCTGGACTACATTG'3 |
|  | 5'GCAAACATATGTACACGGTTCTGG'3 |
| *RAmy1A* | 5'GCGCCTGGTGTCAATCAGAA'3 |
|  | 5'CGGATCGGATACAGCTCGTTG'3 |
| *RAmy3D* | 5'TGCACGGCAAGGACTACAGC'3 |
|  | 5'CCAACGGTTACAAACTGCGTGA'3 |

**Supplemental Table**

**Table S1.** Primers used for quantitative RT-PCR.
