# Supplementary material for: The rice GERMINATION DEFECTIVE 1, encoding a B3 domain transcriptional repressor, regulates seed germination and seedling development by integrating GA and carbohydrate metabolism
Source: Plant J. 2013 May 13;75(3):403–16. doi: 10.1111/tpj.12209 (PMC3813988; doi:10.1111/tpj.12209)
Supplement: Supplementary file 5 [file tpj0075-0403-sd5.docx]

**Supporting Information**

**Figure S1.** Gross morphology of flowers in wild type and *gd1* mutant. Numbered arrows indicate stamens in wild type (a) and *gd1* mutant (b and c). Arrowhead indicates abnormal stigma in *gd1* mutant (b). Sg, stigma; St, stamen. Arrowheads indicate abnormal additional organs (d,e). Stamen and stigma are occasionally fused in *gd1*(f). Scale bar = 1 mm.

**Figure S2.** Cloning of the *GD1* gene. (a) Genomic structure of *GD1* and the T-DNA insertion site in *gd1*. Closed boxes indicate the exons and lines between boxes represent introns. T-DNA was inserted into the eleventh exon. P1, P2, and P4 indicate the position of primers used for co-segregation test. (b) Alignment of the VAL amino acid sequences from rice and *Arabidopsis*. Dark and gray shading indicate the conserved amino acids. The positions of conserved domain are indicated on top of the sequences. (c) Comparison of *GD1* transcript levels between the wild type and *gd1* plants by RT-PCR. *OsActin1* was used as an internal standard. All PCR reactions were amplified with thirty cycles.

**Figure S3.** Expression analysis of GA synthesis and inactivation genes in 4-month-old WT and *gd1* plants by quantitative RT-PCR. Expression levels of each sample were normalized to that of an internal control. Gene expression levels in WT were set as 1.0. Error bars indicate ±SD of three biological replicates. (*) P ≤ 0.05, (**) P ≤ 0.01.

**Table S1.** Primers used for quantitative RT-PCR.
